# Annotations

**Published:** 1910-10-22

**Authors:** 


### Annotations.

### Parliamentary.

An interesting situation was created at the Revision Court for the City of London voters' lists by the claims of several students in residence at St. Bartholomew's Hospital. The leader of those seeking enfranchisement was Mr. Gibson, who seems to have presented his case with much ability. It was proved to the satisfaction of the Revising Barrister (Mr. W. F. Webster) that the claimants all occupied rooms for which a certain rent was paid; and that during their absence the authorities had no power to assign the rooms to other students. As the gentlemen for whom votes were claimed were of full age and had been in occupation for three years, the claims were allowed to stand. From the fact that the Liberal agent acted as an objector to Mr. Gibson's claim, it would seem that in his case at least his political opinions are Conservative. Aside altogether from the party attitude, it seems right and reasonable that medical students nearing qualification should have votes if they can fulfil the conditions imposed by the registration laws; for there are few, indeed, among young men who can have at first hand knowledge of such problems as degeneracy, overcrowding, alcoholism, and other social evils as those whose work lies among the out-patients of a large town hospital.

### The War Office and the St. John Ambulance Association.

Some three months ago attention was prominently drawn in our columns (The Hospital, July 23, 1910, p. 485) to the impasse, in respect of the Voluntary Aid Detachments of the Territorial Medical Service, between the War Office and the St. John Ambulance Association. It was then shown that the latter important organisation had declined to become responsible for the trouble and expense with which the former were, through the County Associations, endeavouring to saddle it; and that the juvenile Red Cross Society, *sans* funds, prestige, and every other resource, was about to shoulder responsibilities which the authorities at Clerkenwell Gate were too far-sighted to accept the burden of. That much negotiation and strenuous efforts to convince the St. John Ambulance Association were in process is evident from a study of the very ambiguous and non-committal replies, in the House of Lords, of the Under-Secretary for War, to questions by the Earl of Dartmouth and others. Now it is clear that an arrangement, if not a settlement, has been arrived at, for the St. John Ambulance Association has formed a Territorial Branch, the secretary of which is Mr. P. G. Darvil-Smith, and has issued instructions for the formation of two classes of local bodies for service with the Territorial Force in war. These bodies will be called the St. John Ambulance Brigade Companies and the St. John Ambulance County Companies respectively. The former will be formed by the St. John Ambulance Brigade under Brigade orders; the latter by the Territorial Branch of the St. John Ambulance Association, but will be able, if they desire, to join the Brigade. No proposal to form a county company will be approved, unless the local Territorial Association has sanctioned it. It does not transpire what *quid pro quo* or concession the St. John Association is to receive for thus abandoning its very prudent attitude of hesitation; nor does it appear from the literature of the Association exactly what happens to the Voluntary Aid Detachments of the Red Cross Society. It is evident that there is a possibility here of over-lapping, not to say of acute rivalry, between these two organisations, for the scheme of the new County Companies is practically the same as that of the already formed Aid Detachments. The St. John Ambulance Association has changed its mind about this matter; nevertheless, we retain the opinion expressed three months ago that, as measures of national protection, these companies and detachments are illusory and untrustworthy. Meanwhile, it may be added that The Hospital is not unmindful or careless of the past glories of the Order of St. John of Jerusalem; and that the gentleman who writes a letter to the *Lancet* suggesting the contrary is basing his conclusion upon a misquotation of what The Hospital said.

### Subdivision of Medical Labour.

The new clinical medicine laboratory of the Edinburgh Royal Infirmary was opened on October 13, Professor Rankine presiding, the ceremony being performed by Sir Clifford Allbutt. The laboratory is provided by an anonymous donor, and a grant in aid of upkeep has been obtained from the Carnegie trustees. Sir Clifford Allbutt, who paid a tribute to Edinburgh M.D.s as practitioners, opines that the establishment of this laboratory forms a step towards the integration of pathological research with clinical medicine—a step calculated to remove the mischievous feud which for the last two thousand years had existed between the active practising physician and the pathologist. The ranks of the profession are not yet recruited solely from exceptional men, and without doubt one of exceptional powers and application is required to spend his evening hours taking opsonic indices, or recording pulse tracings, or conducting the Wassermann reaction after a heavy morning round of visits and an afternoon's operating at his local infirmary, with of course the possibility of a night call to be always remembered. Frankly, such comprehensive study of a case is only possible to those in the leisured position of junior metropolitan consultants, and we regret that although Sir Clifford Allbutt's counsel (like Sir Almroth Wright's at the Royal Society of Medicine last spring) sounds well, yet it was ill-suited to a North British audience in running counter to the maxim of Mr. Barrie's heroine in "What Every Woman Knows"—"Be prahctical!" A pure physiologist, we observe, explained the use of the apparatus of the laboratory.

